# Bone Turnover Markers Changes Induced by Plateletpheresis May Be Minimized with Oral Supplementation of Calcium, Minerals, and Vitamin D before the Procedures: A Non-Randomized, Controlled Study

**DOI:** 10.3390/jcm12010281

**Published:** 2022-12-29

**Authors:** Edgar Barrientos-Galeana, Mari Cruz Tolentino-Dolores, Rosa María Morales-Hernández, Reyna Sámano, Gabriela Chico-Barba, Emmanuel Fernández-Sánchez, Lizbeth Jazmín Zamora-Sánchez, Alma Delia Alonso-López, Heriberto López-Martínez, Tania Alba-Rosales, Sergio Arturo Sánchez-Guerrero

**Affiliations:** 1 Nutrition and Bioprogramming Coordination, Instituto Nacional de Perinatología, Mexico City 11000, Mexico; 2 Blood Bank Department, Instituto Nacional de Cancerología, Mexico City 14080, Mexico; 3 Regulatory Direction, Centro Nacional de la Transfusión Sanguínea, Mexico City 07700, Mexico

**Keywords:** apheresis, magnesium, copper, zinc, citrate infusion, hypocalcemia

## Abstract

Apheresis allows the collection of specific blood components but changes serum calcium (Ca), magnesium (Mg), copper (Cu), zinc (Zn), and hormones involved in bone metabolism due to citrate infusion. We assessed the effect of oral supplementation of calcium, vitamin D, and minerals as pills or an enriched diet before plateletpheresis donation on levels of divalent cations, hormones, and bone turnover markers that may prevent metabolic changes. Methods: Non-randomized controlled study including 134 donors. Serum parathyroid hormone (PTH), Ca, Mg, Zn, Cu, osteocalcin (OC), vitamin D, and type-1 collagen C-terminal telopeptide (CTX-1) levels were measured at baseline and post-procedure. Donors were divided into four groups: supplemented with calcium carbonate and vitamin D (cal + vitd); those receiving calcium, minerals, and vitamin D (cal + vitd + min); those receiving a calcium-rich diet (diet) and a control group (control). Results: PTH levels increased >1-fold, whereas tCa, tMg, Zn, Cu, iCa, iMg, and vitamin D levels decreased immediately after apheresis amongst donors of any group; when these levels were measured two weeks later, donors in the calcium-vitd and cal + vitd + min groups returned to basal values; donors in the cal + vitd + min group were the only group that kept their levels of OC and CTX unchanged at the different study times. Conclusions: Bone turnover markers changes induced by plateletpheresis may be minimized with oral supplementation of calcium, minerals, and vitamin D two days before the procedures.

## 1. Introduction

The term apheresis comes from the Greek word aphaeresis, which means “taking away”, and has been used to describe those medical procedures involving the mechanical separation of whole blood into its components with the specific removal of one of these components [1]. It is well known that apheresis is an efficient method to collect platelets, leukocytes, plasma, or stem cells, with some advantages like the collection of standardized and high-quality blood products and a higher collection frequency [2] and the benefit of reduced recipient exposures. This may be one of the reasons why plateletpheresis is now frequently used for transfusion. 

Safety issues for donors have been a concern throughout the apheresis donation program, as demonstrated in a recently published paper [3] that assessed the safety of triple plateletpheresis. A citrate infusion is used during apheresis and causes acute metabolic changes, resulting in the chelation of divalent cations, especially serum magnesium (Mg), total calcium (tCa), and ionized calcium (iCa). The latter is the physiologically active form of calcium [4]. Hypocalcemia and hypomagnesemia are usually manifested by mild perioral or acral paresthesia, whereas more severe citrate toxicity may produce carpopedal spasm, nausea, and vomiting, including frank tetany [5,6,7,8]. Acid-citrate-dextrose (ACD-A) has been the most commonly used formula for apheresis procedures during the last 40 years [9,10] mainly due to its low cost and fast metabolism (T½ = 30 to 60 min) [4,11,12]. However, the time that clinical manifestations remain in the donor is unknown. 

For this reason, alternatives have been sought to reduce the adverse effects and safeguard the health of donors. Mineral supplementation has been used in prior apheresis processes or during oral and intravenous extraction processes with calcium (Ca), Mg, potassium, or vitamin D [13,14,15,16]. Unfortunately, only the frequency of adverse reactions decreased, and metabolic changes remained.

Whereas most publications refer to immediate adverse reactions, long-term adverse effects, particularly on mineral homeostasis and bone metabolism, have scarcely been investigated [2,17,18]. Furthermore, the chelation of other divalent cations, such as zinc (Zn) and copper (Cu), has not previously been assessed. Regarding acute citrate-related adverse reactions to plateletpheresis, their frequency ranges from 0.12% to 58% [5,6,9,10]. Most countries limit the number of plateletpheresis procedures to 24 or 26 per patient per year [3]. 

This study aimed to assess the effect of oral supplementation of calcium, vitamin D, and minerals as pills or an enriched diet before plateletpheresis donation on the levels of divalent cations, hormones, and bone turnover markers (BTMs), which may minimize metabolic changes.

## 2. Materials and Methods

### 2.1. Study Population 

This was a controlled, before-and-after, non-randomized study, including 137 healthy donors who attended the Instituto Nacional de Cancerología in Mexico City. We included men and non-pregnant or non-lactating women, 18–64 years of age, with a platelet count greater than 200 × 10^3^/µL and adequate venous access for the procedure. The apheresis donors were unpaid, replacement (family), and altruistic, according to the Mexican donation guidelines [19], based on international guidelines. In addition, they completed a medical evaluation, including a clinical questionnaire, physical examination, and self-exclusion form, performed by a licensed physician according to the Mexican Blood Bank Official Norm [19]. We excluded those intended participants taking any vitamins or minerals before being incorporated into this study. We also eliminated donors whose infectious serology testing came out positive or who did not adhere to the assigned group.

Our institutional ethical and research committees approved the study protocol, and written informed consent was obtained from every donor before plateletpheresis. We used a systematic allocation method based on the order in which patients presented to our study for convenience [20]. 

### 2.2. Blood Samples 

All plateletphereses were performed between 7:00 and 11:00 AM using the Amicus separator (Fenwal, Deerfield, IL, USA) and the Trima Accel (Terumo BCT, Denver, CO, USA). ACD-A anticoagulant was dripping during the whole apheresis procedure (which lasted 70 min on average) at a whole blood to anticoagulant ratio of 10:1 and a mean flow rate of 60 mL/min, according to our standard operating procedure manual. Blood samples were drawn from the system’s piggy bag both at the beginning (time-1) of the procedure and 10’ after finishing plateletpheresis (time-2). In addition, a third sample was drawn 15 days later from the participating donors (time-3). We used the trace-free Vacutainer tubes (Becton-Dickinson, Franklin Lakes, NJ) for this performance. All sera samples were frozen at −70 °C until analytic tests were performed.

### 2.3. Intervention 

Once included in the study, donors were divided into four groups: group 1 (control), donors received no supplementation; group 2 (diet), donors were given a calcium-rich diet, which was designed and assigned by specialized nutritionists according to their nutritional requirements, and was equivalent to the supplemented minerals in groups 3 and 4; group 3 (cal + vitd), donors received calcium carbonate (600 mg) plus vitamin D3 (200 IU) pills twice a day; and group 4 (cal + vitd + min), donors received calcium carbonate (600 mg), minerals (Mg [40 mg], Zn [7.5 mg] and Cu [1 mg]) and vitamin D3 (200 IU) pills twice a day. Interventions (diet or pills) started 48 h before plateletpheresis The intervention period was based on the absorption time of Ca, which can be from 7 to 26 h [21]; for the rest of the minerals, the absorption time is unknown. Dietetic intake evaluation in all participants was assessed using a food frequency questionnaire validated for the Mexican population (SNUT 2003). The energy adequacy percentage was calculated based on the recommended energy intake per sex and age, and the total protein percentage was calculated based on the total energy intake.

### 2.4. Biomarkers Assessment 

Measurements were performed with the following techniques:

- Total calcium and magnesium (tCa, tMg) by colorimetric assay (Vitros, Ortho Clinical Diagnostics, Raritan, NJ, USA);
- Ionized calcium and magnesium (iCa, iMg) by an ion-selective electrode (Stat Profile Prime Plus, Nova Biomedical, Waltham, MA, USA); copper and zinc (Cu, Zn) by atomic absorption spectrophotometry (AAnalyst 400 Perkin Elmer, Wellesley, MA, USA);
- Collagen type-1 C-terminal telopeptide (CTX-1) (DRG International Inc., Springfield, NJ, USA), osteocalcin (OC) and parathyroid hormone (PTH) (Immulite 1000, Siemens, Munich, Germany) and 25-OH Vitamin D, Architect i1000 SR, Abbott Diagnostics (Chicago, IL, USA) by enzyme immunoassay.

### 2.5. Statistical Analysis 

The sample size was calculated based on the formula of paired means; the expected difference in the PTH values before and after the intervention was 30 units. A confidence level of 95% and a power of 80% were determined. Statistical analyses included independent Student’s *t*-tests or ANOVA for comparison between groups at different times in the study, with normal distribution and Mann–Whitney U or Kruskal–Wallis tests for non-normal distributions. Paired Student’s *t*, Wilcoxon and Friedman’s tests were used to compare values within the same group at different times. We then performed stratified analyses according to the median age of the sample (≤31 vs. >32 years) and BMI categories (normal weight vs. overweight/obesity); a *p*-value less than 0.05 was considered significant. 

## 3. Results

### 3.1. General Characteristics

**Figure 1:**
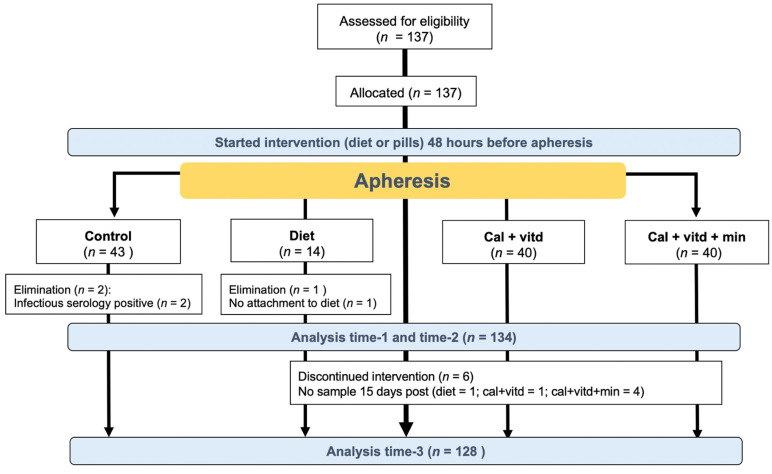
Flowchart of the studied population. Control: no supplementation; Diet: diet; cal + vitd: calcium carbonate + vitamin D; cal + vitd + min: calcium carbonate, minerals + vitamin D. Blood samples were taken at the beginning (time-1); after finishing the plateletpheresis (time-2); and fifteen days later from the participating donors (time-3).

One hundred and thirty-seven consecutive donors entered the study from June 2016 through June 2018; however, two were excluded due to positive results in their infectious serologic tests and a third due to a lack of adherence to diet, as shown in Figure 1. 

After elimination, 134 donors were included in the study, and 56% of the population were males. The general characteristics of our donor population can be seen in Table 1. There were no statistically significant differences between groups regarding the number of platelets at the end of donation, infused citrate volume, or length of the procedures. However, we found that our control group of donors was significantly older when compared to the other groups; the data did not show differences associated with age or gender. Additionally, the diet group was the smallest; thus, we did not discuss this group’s results, nor compare it to the other groups.

### 3.2. Nutrients Intake

Table 2 shows the nutrient consumption in every group of donors. As can be seen, there were no statistically significant differences between them. However, we must mention that nutrient intake in the studied sample did not satisfy the recommended daily intake for several nutrients.

### 3.3. Metabolic Changes

**Figure 2:**
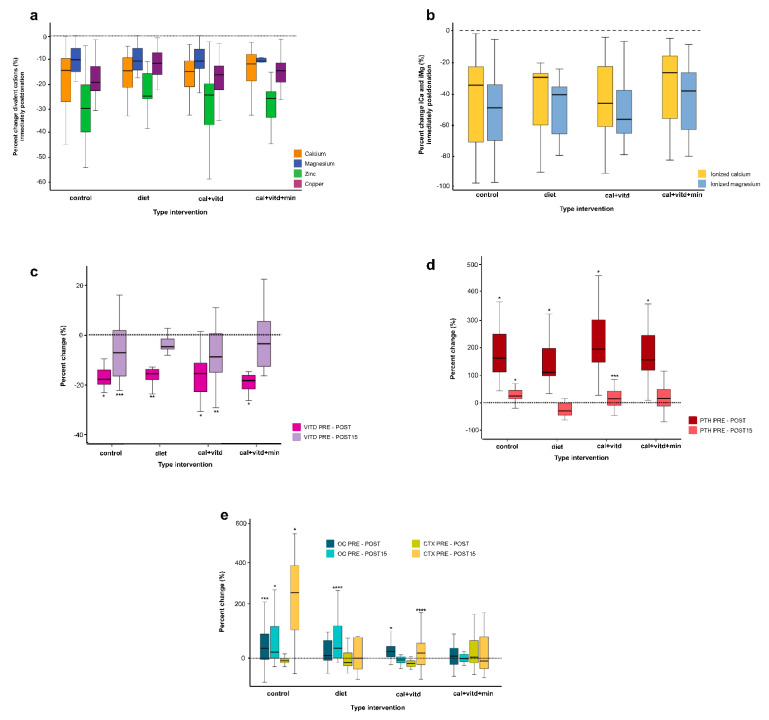
Effect of citrate on the plasma levels of total divalent cations (**a**), ionized cations (**b**), hormones (**c**,**d**), and bone turnover markers (**e**) in plateletpheresis donors at different times of the study. VITD: 25-OH Vitamin D, PTH: Parathyroid hormone; OC: Osteocalcin; CTX: C-terminal telopeptide of collagen type; CONTROL: No supplementation; DIET: Calcium-rich diet; CAL-VITD: Supplementation with calcium carbonate + vitamin D; CAL + VITD + MIN: Supplemented with calcium carbonate + minerals + vitamin D. The dotted line represents the basal state. PRE-POST: changes in serum post-donation levels compared to pre-donation levels. PRE-POST15: changes in serum levels 15 days after plateletpheresis compared to pre-donation levels. The results show statistical differences by Wilcoxon. *p ≤ 0.001, ** *p* ≤ 0.005, ***p ≤ 0.010, **** *p* ≤ 0.050.

Regarding the metabolic changes shown in Table 3, as a result of plateletpheresis, tCa, tMg, iCa, iMg Zn, and Cu levels decreased statistically significantly and even reached values below reference ranges (Figure 2a,b); 15 days later, Zn and Cu returned to basal values in all groups.

In the stratified analyses by age group and BMI category, we observed statistical differences in Cu, PTH, OC, CTX, and Vitamin D. Nevertheless, the sample size of each category within the groups was small. 

The vitamin D values decreased significantly immediately after plateletpheresis; in contrast, PTH levels increased by over 100% of their baseline values independent of the intervention group. Furthermore, for both hormones, the diet and cal + vitd + min groups recovered their baseline vitamin D and PTH values two weeks later (Table 3 and Figure 2c,d). 

Finally, Figure 2e shows the BTM changes associated with plateletpheresis. The control group showed OC and CTX level increases by over 200% of the basal values even on day 15 after plateletpheresis (Table 3). The cal + vitd + min group was the only group that did not show statistical changes in BTMs for the three evaluated times (Table 3 and Figure 2e).

In comparison with the control group as a reference, statistical differences were observed in PHT (diet *p* < 0.001; cal + vitd *p*= 0.003; cal + vitd + min *p* < 0.001) and for CTX (diet *p* = 0.019; cal + vitd *p* = 0.038; cal + vitd + min *p* < 0.017).

## 4. Discussion

In our study, all cations (tCa, tMg, iCa, iMg, Zn, and Cu) and vitamin D levels significantly decreased immediately after apheresis donation, in contrast to PTH, which increased more than 100%. These changes were observed for all types of intervention. However, we interestingly found a trend to accelerate homeostasis in those donors receiving oral supplementation of cal + vitd + min, as assessed by PTH, OC and CTX-1 in the following 15 days post plateletpheresis compared with the control and diet groups. 

In a previous study [23] of 105 donors, we found that plateletpheresis increased levels of PTH due to calcium chelation by citrate. In addition, we also demonstrated that other cations (Mg, Cu and Zn) decreased significantly. Therefore, we decided to investigate if we could prevent this phenomenon or mitigate the BTM changes. We did it by following a cohort of plateletpheresis donors whom we treated with any of the following: calcium-rich diet or supplementation with prophylactic intake of different minerals (cal + vitd or cal + vitd + min) compared to a control group of donors in order to assure that infusion of citrate during plateletpheresis sessions will not become a risk factor to develop osteopenia and osteoporosis [2,17,18]. 

According to our results, we suggest that it is worthwhile to reassess the number of maximum procedures in a year. In Mexico, the current norm for blood banks states that a 15-day interval between plateletpheresis treatments must be observed [19]. However, we are not quite sure that permitting a maximum of 24 to 26 procedures in a year is safe enough for donors who undergo apheresis for several decades. Due to the evolution of medicine and the discovery of new anticoagulants in clinical practice, we think that the time has come to develop other options besides citrate (which has been employed in clinical practice for one century) [24] to be used during the great variety of apheresis protocols. We and other authors [2,18,25] believe that metabolic changes may occur in donors (and patients) undergoing plasmapheresis, plasma exchange, leukapheresis, or stem cell collection. Heparin alone or in combination with citrate has been used in some procedures. Even though it has been effective, some adverse effects, such as bleeding and heparin-induced thrombocytopenia, have been reported [4]. Therefore, it is important to keep in mind other strategies, such as the preventive measures suggested and formulated here to maintain donors’ health. This preventive strategy was already suggested [15,16,26], even though this recommendation has been questioned [27] and there has been controversy around the low–null risk of fractures by subsequent donations [28]. Although some studies in the apheresis donor population assess bone mineral changes [18,29] and oral supplementation [15,16], we do not consider them comparable to our current study due to several methodologic differences, such as nutritional supplementation, time intervals and statistical analysis.

It is evident, though, that additional supplementation of Mg and vitamin D can help to prevent such adverse effects as a consequence of the procedure. The importance of studying iMg is due to the similarity of manifestations between hypomagnesemia and hypocalcemia, as well as the relationship with disorders in the metabolism of vitamin D and other physiological and metabolic alterations [8,30,31,32]. Our results showed that both Cu and Zn were chelated by citrate, decreasing by between 10 and 30% of their serum levels (Figure 2a). As Zn is implicated in several metabolic processes, we are concerned mainly because, in previous studies, Zn deficiency was observed in 50% of a sample in a Mexican population [33,34,35]. Furthermore, recent studies examined zinc´s relevance in preventing and reversing osteoporosis, acting as a local regulator of bone cells preventing PTH-induced bone resorption. Moreover, according to our findings, essential trace elements, such as Cu (which may also be chelated by citrate), are required along with Zn to maintain healthy bone tissue [36,37,38]. 

On the other hand, the increase in PTH occurs when the parathyroid glands respond to a reduction in the serum levels of ionized calcium. Our results show that, in the cal + vitd group of donors, their iCa and iMg levels decreased by up to 53% compared with their basal levels (Figure 2b). Consequently, PTH concentrations rise (Figure 2d) while bone resorption increases (Figure 2e). 

It has been postulated that women are prone to experiencing acute citrate reactions. Unfortunately, most published papers assessing adverse reactions to apheresis focus on venous access, circulatory problems, and immediate citrate toxicity [3,6]. We believe it is important to prevent potential long-term adverse effects [2,18,39,40,41]. Those few papers addressing the metabolic changes secondary to apheresis in this scenario show that both PTH and cation levels return to normal within 24 h [2,42]. However, the long-term effect of the repetitive stimulus of cation chelation on PTH in those persons who donate platelets by apheresis several times a year for their adult lives is still an unanswered question. 

At this point, we must recognize that our donor population had a poor nutrient intake that did not meet calcium and other mineral requirements [33]. We know that these phenomena may occur in other countries (especially in non-developed countries). Furthermore, it has been recently stated that elevated PTH levels may increase the risk of cardiovascular diseases and may also be linked to metabolic syndrome [31,43,44]. Therefore, there are still important questions to be addressed to make apheresis safer and more comfortable for donors, patients, and health workers taking care of them. In addition, our findings regarding the decreases in Cu and Zn levels deserve further investigation to clarify if such changes could favor chronic damage to bone health.

We also assessed our cohort of donors’ changes in BTMs. It is important to point out the significant shifts in OC and CTX levels, mainly in the control group, which did not return to basal values even two weeks later; similar results were described at two hours [18] and 24 h post-procedure [2]. Although the process does not allow monitoring of all the aforementioned laboratory parameters described during the donation process, it is possible to look for alternatives to mitigate or counteract the effect of citrate. 

The main limitation of our study was the small number of participants, especially in the diet group, because they did not adhere to the dietary intervention according to the adherence assessment with the food frequency questionnaire. The differences observed in the stratified analyses for some metabolic changes and BTMs should also be cautiously interpreted, because the sample size in each stratum was too small; therefore, more studies with larger sample sizes are needed. It is noteworthy to say that this was challenging work because of the return of donors 15 days after apheresis. It is also a strength to have samples from the three moments in the study, because the works of other collaborators only analyze pre- and post-apheresis.

## 5. Conclusions

In all intervention groups, the plateletpheresis donors showed an immediate post-donation decrease in Ca, Mg, iCa, iMg, Zn, Cu, and vitamin D and an increase in PTH. Bone turnover marker (OC and CTX) changes may be minimized immediately and after 15 days, minimized by supplementation with cal + vitd + min starting two days before the procedures. More studies on clinical outcomes are needed to support this type of intervention.

## Figures and Tables

**Table 1:** General characteristics of donors by study group.

	Study Groups				
	CONTROL	DIET	CAL + VITD	CAL + VITD + MIN	*p*-Value
N	42	13	39	40	
Males	26 (62) ^a^	6 (46) ^a^	24 (61) ^a^	19 (48) ^a^	------
Females	16 (38) ^a^	7 (54) ^a^	15 (39) ^a^	21 (52) ^a^	------
Age	37 (31–45) ^b^	31 (23–39) ^b^	30 (24–37) ^b^	29 (23–32) ^b^	0.001 ^c^
BMI	29 (4)	27 (4)	27 (4)	27 (4)	0.019 ^d^
Bone fractures (events/life)	2 (5)	0	0	0	0.221 ^d^
Alcohol intake (cup/day)	11 (26)	1 (8)	2 (5)	2 (5)	0.587 ^d^
Platelets PRE × 10^3^/µL	287 (47)	290 (49)	260 (43)	285 (50)	0.035 ^d^
Platelets POST × 10^3^/µL	179 (28)	190 (33)	166 (26)	178 (34)	0.088 ^d^
Apheresis duration (min) ^b^	72 (64–81)	70 (64–80)	71 (63–79)	70 (63–81)	0.595 ^c^
Infused citrate (mL)	293 (39)	283 (37)	287 (43)	281 (44)	0.624 ^d^
Previous donations	1 (0–2)	2 (2–4)	1 (1–1)	1 (1–2)	0.065 ^d^

Control: no supplementation; Diet: diet; cal + vitd: calcium carbonate + vitamin D; cal + vitd + min: calcium carbonate, minerals + vitamin D. Results shown as mean ± SD; ^a^: N (%); ^b^: median (P_25_–P_75_); ^c^: Kruskal-Wallis; ^d^: ANOVA; PRE: predonation; POST: postdonation.

**Table 2:** Nutrients intake in donors according to the group of study.

	Study Group					
Nutrients per Day	CONTROL	DIET	CAL + VITD	CAL + VITD + MIN	RDIs ^b^	*p*
	median (P_25_–P_75_)	median (P_25_–P_75_)	median (P_25_–P_75_)	median (P_25_–P_75_)		
Energy (Kcal)	2302 (1778–2730)	2108 (1735–2471)	1995 (1752–2356)	2053 (1698–2632)	2100F–3100M	0.641 ^c^
Energy adequacy (%)	80 (66–101)	83 (64–105)	67 (57–100)	80 (62–103)	100	0.641 ^c^
Total fat (g)	84 (63–105)	71 (65–91)	79 (56–100)	80 (60–100)	210–310	0.575 ^c^
Total Proteins (%)	14 (12–15)	15 (13–16)	14 (13–16)	14 (12–16)	10–15% (Kcal)	0.986 ^c^
Carbohydrates (g) ^a^	282 (94)	271 (97)	266 (110)	270 (107)	630–930	0.918 ^d^
Raw Fiber (g) ^a^	6 (3)	6 (2)	6 (2)	6 (2)	18–24	0.377 ^d^
Calcium (mg)	606 (463–821)	679 (436.75–734.36)	612 (484–877)	666 (493–824)	1200	0.997 ^c^
Magnesium (mg)	322 (240–385)	298 (270–383)	286 (232–365)	303 (245–383)	250F–340M	0.880 ^c^
Zinc (mg)	16 (13–20)	17 (12–20)	16 (13–20)	17 (12–25)	11F–12N	0.483 ^c^
Copper (mg)	2 (1–3)	2 (1–3)	2 (2–3)	3 (2–4)	0.9	0.122 ^c^
Sodium (mg)	1824 (1355–2369)	1728 (1499–2208)	1482 (1270–2057)	1515 (1160–2101)	1600	0.302 ^c^
Phosphorus (mg)	1233 (907–1439)	1128 (1004–1405)	1140 (909–1489)	1194 (880–1594)	580F–700M	0.988 ^c^
Vitamin C (mg)	126 (68–209)	179 (110–299)	157 (106–196)	118 (91–197)	57F–84M	0.256 ^c^
Vitamin D (UI)	111 (69–240)	138 (88–185)	165 (105–279)	142 (91–249)	525–775	0.125 ^c^

Control: no supplementation; Diet: diet; cal + vitd: calcium carbonate + vitamin D; cal + vitd + min: calcium carbonate, minerals + vitamin D; ^a^: mean ± SD; ^b^: RDIs: Recommended daily intake suggested for Mexican population; ^c^: Kruskal–Wallis; ^d^: ANOVA; F: females; M: males.

**Table 3:** Metabolic changes post-donation in plateletpheresis donors.

Metabolic Changes								
Type of Intervention	Biomarker	Time 1	Time 2	Time 3	*p*-Value			
		Pre-Donation	Post-Donation	Post-Donation 15 Days	Time 1 vs. Time 2 ^a^	Time 1 vs. Time 3 ^a^	Time 2 vs. Time 3 ^a^	All Times ^b^
Control	Ca ^c^	9.7 (9.4–10.0)	8.2 (6.9–8.7)	nd	<0.001	na	na	na
	iCa ^d^	1.14 (1.06–1.17)	0.70 (0.34–0.87)	nd	<0.001	na	na	na
	Mg ^c^	2.1 (2.0–2.2)	1.9 (1.8–2.0)	nd	<0.001	na	na	na
	iMg ^d^	0.54 (0.43–0.55)	0.25 (0.15–0.33)	nd	<0.001	na	na	na
	Zn ^e^	96.4 (83.5–126.9)	70.1 (63.9–71.9)	94.3 (84.3–107.8)	<0.001	0.534	0.003	<0.001
	Cu ^e^	102.2 (88.1–117.3)	85.9 (74.3–94.7)	102.5 (92.4–118.1)	<0.001	0.794	0.003	<0.001
	PTH ^f^	41.0 (35.50–44.10)	97.90 (78.80–138)	50.0 (42.40–64.60)	<0.001	<0.001	<0.001	<0.001
	VIT D ^g^	22.55 (17.5–26.9)	18.55 (14.72–21.6)	21.50 (18.40–27.25)	<0.001	0.010	<0.001	<0.001
	OC ^g^	3.2 (1.5–6.38)	4.1 (2.1–7.1)	5.59 (4.24–7.3)	0.010	0.001	0.469	0.013
	CTX ^g^	0.24 (0.20–0.28)	0.21 (0.19–0.27)	0.86 (0.42–1.25)	<0.001	<0.001	<0.001	<0.001
Diet	Ca ^c^	9.8 (9.5–9.9)	8.3 (7.9–9.0)	nd	0.001	na	na	na
	iCa ^d^	1.19 (1.15–1.21)	0.82 (0.48–0.87)	nd	0.001	na	na	na
	Mg ^c^	2.0 (1.9–2.2)	1.9 (1.8–2.0)	nd	0.002	na	na	na
	iMg ^d^	0.54 (0.50–0.59)	0.31 (0.21–0.35)	nd	0.001	na	na	na
	Zn ^e^	89.0 (80.4–99.5)	71.4 (66.3–76.4)	83.8 (72.6–89.0)	0.001	0.893	0.043	0.001
	Cu ^e^	120.5 (112.1–133.6)	108.9 (101.0–116.0)	103.9 (96.2–107.3)	0.001	0.008	0.173	0.001
	PTH ^f^	41.1 (27.7–48.5)	88.1 (79–123)	25.7 (22.72–37.25)	0.001	0.015	0.002	<0.001
	VIT D ^g^	20.5 (17.2–25.0)	16.9 (13.9–21.9)	19.90 (15.20–23.75)	0.001	0.173	0.008	0.001
	OC ^g^	5.7 (3.1–13.7)	6.0 (3.7–13.25)	5.6 (4.1–10.77)	0.507	0.028	0.575	0.741
	CTX ^g^	0.22 (0.16–0.51)	0.37 (0.16–0.42)	0.37 (0.10–1.04)	0.600	0.445	0.241	0.497
Cal + vitd	Ca ^c^	9.5 (9.3–10.0)	8.2 (7.7–8.6)	nd	<0.001	na	na	na
	iCa ^d^	1.17 (1.05–1.19)	0.60 (0.42–0.85)	nd	<0.001	na	na	na
	Mg ^c^	2.0 (1.9–2.1)	1.8 (1.7–1.9)	nd	<0.001	na	na	na
	iMg ^d^	0.53 (0.49–0.58)	0.21 (0.16–0.34)	nd	<0.001	na	na	na
	Zn ^e^	93.3 (84.6–104.2)	67.2 (58.7–77.9)	90.1 (81.6–100.2)	<0.001	0.447	<0.001	<0.001
	Cu ^e^	93.1 (78.6- 114.5)	78.2 (66.7–93.6)	88.0 (75.8–103.9)	<0.001	0.586	0.006	<0.001
	PTH ^f^	34.1 (26.5–43.7)	101 (77.1–141.0)	33.35 (25.37–50.15)	<0.001	0.015	<0.001	<0.001
	VIT D ^g^	22.4 (17.6–25.40)	18.0 (14.4–21.8)	20.60 (16.17–24.77)	<0.001	0.002	0.012	<0.001
	OC ^g^	6.8 (4.6–10.0)	8.9 (5.6–12.1)	5.91 (4.9–8.9)	0.001	0.466	0.008	<0.001
	CTX ^g^	0.38 (0.30–0.55)	0.32 (0.24–0.42)	0.42 (0.22–0.92)	<0.001	0.026	0.006	0.007
Cal + vitd + min	Ca ^c^	9.9 (9.5–10.3)	8.6 (8.1–9.0)	nd	<0.001	na	na	na
	iCa ^d^	1.15 (1.1–1.18)	0.79 (0.50–0.95)	nd	<0.001	na	na	na
	Mg ^c^	2.0 (2.0–2.1	1.9 (1.8–2.0)	nd	<0.001	na	na	na
	iMg ^d^	0.54 (0.48–0.56)	0.29 (0.19–0.36)	nd	<0.001	na	na	na
	Zn ^e^	98.9 (91.1–106.9)	74.9 (68.7–78.3)	98.3 (86.7–107.7)	<0.001	0.219	<0.001	<0.001
	Cu ^e^	110.9 (884.3–126.1)	94.6 (69.4–108.0)	89.3 (78.1–97.1)	<0.001	0.859	0.086	0.012
	PTH ^f^	32.8 (25.1–48.8)	91.3 (68.72–133.5)	36.90 (27.52–46.90)	<0.001	0.083	<0.001	<0.001
	VIT D ^g^	19.0 (14.52–23.6)	15.4 (11.6–19.1)	18.90 (17.67–24.80)	<0.001	0.221	0.001	<0.001
	OC ^g^	5.4 (3.4–8.0)	6.4 (3.6–8.6)	5.9 (5.49–7.72)	0.267	0.984	0.687	0.692
	CTX ^g^	0.40 (0.23–0.70)	0.46 (0.35–0.64)	0.40 (0.18–0.71)	0.648	0.451	0.696	0.756

The results show medians (P_25_–P_75_); ^a^: Wilcoxon test; ^b^: Friedman test; ^c^: mg/dL; ^d^: mmol/L; ^e^: μg/dL; ^f^: pg/mL; ^g^: ng/mL: nd: undetermined due to difficulties in collection and preservation of samples, and equipment specifications; na: not applicable. Reference values: iCa: 1.17–1.51 mmol/L; iMg: 0.44–0.60 mmol/L [16]; Zn: >65 μg/dL; Cu: >70 μg/dL μg/dL [22]; Ca: 9.0–11.0 mg/dL; Mg: 1.7–2.7 mg/dL; PTH: 15–65 pg/mL; VITD: 30–60 ng/mL; OC: 1–35 ng/mL; CTX: 0.06–0.35 ng/mL [2].

## Data Availability

The data presented in this study are available on request from the corresponding author. The data are not publicly available due to privacy reasons.

## References

[1] Winters J.L. (2012). Plasma exchange: Concepts, mechanisms, and an overview of the American Society for Apheresis guidelines. Hematol. Am. Soc. Hematol. Educ. Progr..

[2] Amrein K., Katschnig C., Sipurzynski S., Stojakovic T., Lanzer G., Stach E., Pieber T.R., Dobnig H. (2010). Apheresis affects bone and mineral metabolism. Bone.

[3] Heuft H.-G., Moog R., Fischer E.G., Zingsem J., German and Austrian Plateletpheresis Study Group (2013). Donor safety in triple plateletpheresis: Results from the German and Austrian Plateletpheresis Study Group multicenter trial. Transfusion.

[4] Lee G., Arepally G.M. (2012). Anticoagulation techniques in apheresis: From heparin to citrate and beyond. J. Clin. Apher..

[5] Yuan S., Ziman A., Smeltzer B., Lu Q., Goldfinger D. (2010). Moderate and severe adverse events associated with apheresis donations: Incidences and risk factors. Transfusion.

[6] Makar Y.F., Butler M.O., Cockersole G.M., Gabra G., Serevitch J.M. (2002). National audit of citrate toxicity in plateletpheresis donors. Transfus. Med..

[7] Wiltbank T.B., Giordano G.F. (2007). The safety profile of automated collections: An analysis of more than 1 million collections. Transfusion.

[8] Kostov K. (2019). Effects of Magnesium Deficiency on Mechanisms of Insulin Resistance in Type 2 Diabetes: Focusing on the Processes of Insulin Secretion and Signaling. Int. J. Mol. Sci..

[9] Huestis D., Fletcher J., White R., Price M. (1977). Citrate anticoagulants for plateletpheresis. Transfusion.

[10] Van de Meer P. (2012). Apheresis versus whole-blood-derived platelets: Pros and cons. ISBT Sci. Ser..

[11] Davenport A., Tolwani A. (2009). Citrate anticoagulation for continuous renal replacement therapy (CRRT) in patients with acute kidney injury admitted to the intensive care unit. NDT Plus.

[12] Strobl K., Harm S., Weber V., Hartmann J. (2017). The Role of Ionized Calcium and Magnesium in Regional Citrate Anticoagulation and its Impact on Inflammatory Parameters. Int. J. Artif. Organs.

[13] Passos-Coelho J.L., Braine H.G., Wright S.K., Davis J.M., Schepers K.G., Huelskamp A.-M., Clarke B., Noga S.J., Kennedy M.J. (1995). Large-Volume Leukapheresis Using Regional Citrate Anticoagulation to Collect Peripheral Blood Progenitor Cells. J. Hematother..

[14] Haddad S., Leitman S.F., Wesley R.A., Cecco S., Yau Y.Y., Starling J., Rehak N.N., Bolan C.D. (2005). Placebo-controlled study of intravenous magnesium supplementation during large-volume leukapheresis in healthy allogeneic donors. Transfusion.

[15] Weinstein R., Haynes S., Zhao Y., Hickson E., Linden J., Pierre P.S., Ducharme P., Sulmasy P., Graves M., Bailey J.A. (2017). A liquid calcium+vitamin D_3_ supplement is effective prophylaxis against hypocalcemic toxicity during apheresis platelet donation. J. Clin. Apher..

[16] Bolan C.D., Wesley R.A., Yau Y.Y., Cecco S.A., Starling J., Oblitas J.M., Rehak N.N., Leitman S.F. (2003). Randomized placebo-controlled study of oral calcium carbonate administration in plateletpheresis: I. Associations with donor symptoms. Transfusion.

[17] Boot C.L., Luken J.S., can der Burg P.J.M., de Kort W.L.A.M., Koopman M.M.W., Vrielink H., van Schoor N.M., Heijer M.D., Lips P. (2015). Bone density in apheresis donors and whole blood donors. Vox Sang..

[18] Chen Y., Bieglmayer C., Höcker P., Dettke M. (2009). Effect of acute citrate load on markers of bone metabolism in healthy volunteers. Vox Sang..

[19] (2012). Norma Oficial Mexicana NOM-253-SSA1-2012, Para la Disposición de Sangre Humana y Sus Componentes con Fines Terapéuticos.

[20] Sedgwick P.M. (2017). Nonrandomized Trials: Designs and Methodology. Emerg. Themes Epidemiol..

[21] Barger-Lux M.J., Heaney R.P., Recker R.R. (1989). Time course of calcium absorption in humans: Evidence for a colonic component. Calcif. Tissue Res..

[22] Mejía-Rodríguez F., Shamah-Levy T., Villalpando S., García-Guerra A., Méndez-Gómez Humarán I. (2013). Iron, zinc, copper and magnesium deficiencies in Mexican adults from the National Health and Nutrition Survey 2006. Salud Publica Mex..

[23] Guerrero S.A.S., Canto N.P.Z., Dolores M.T., Ibarra A.B., Hernández M.C., Méndez J.R.L., Sánchez L.Z., López A.D.A., Pichón E.R., Gómez L.B. (2013). Hiperparatiroidismo secundario inducido por plaquetoféresis como resultado de la quelación de los cationes divalentes por el citrato. Rev. Mex. Med. Transf..

[24] Mollison P.L. (2000). The introduction of citrate as an anticoagulant for transfusion and of glucose as a red cell preservative. Br. J. Haematol..

[25] Bolan C.D., Greer S.E., Cecco S.A., Oblitas J.M., Rehak N.N., Leitman S.F. (2001). Comprehensive analysis of citrate effects during plateletpheresis in normal donors. Transfusion.

[26] Buchta C., Macher M., Bieglmayer C., Höcker P., Dettke M. (2003). Reduction of adverse citrate reactions during autologous large-volume PBPC apheresis by continuous infusion of calcium-gluconate. Transfusion.

[27] Palfi M., Martinsson L., Sundström K. (2007). Hypocalcemic symptoms during plateletpheresis using the COBE Spectra: A comparison of oral combination of 600mg calcium+300mg magnesium+100IU vitamin D3 vs. a 1000mg calcium in symptomatic donors. Transfus. Apher. Sci..

[28] Grau K., Vasan S.K., Rostgaard K., Bialkowski W., Norda R., Hjalgrim H., Edgren G. (2017). No association between frequent apheresis donation and risk of fractures: A retrospective cohort analysis from S weden. Transfusion.

[29] Bialkowski W., Blank R.D., Zheng C., Gottschall J.L., Papanek P.E. (2018). Impact of frequent apheresis blood donation on bone density: A prospective, longitudinal, randomized, controlled trial. Bone Rep..

[30] Chu C., Zhao W., Zhang Y., Li L., Lu J., Jiang L., Wang C., Jia W. (2016). Low serum magnesium levels are associated with impaired peripheral nerve function in type 2 diabetic patients. Sci. Rep..

[31] Barbagallo M., Dominguez L.J. (2015). Magnesium and Type 2 Diabetes: An Update. Int. J. Diabetes Clin. Res..

[32] Chaudhary D.P., Sharma R., Bansal D.D. (2009). Implications of Magnesium Deficiency in Type 2 Diabetes: A Review. Biol. Trace Elem. Res..

[33] Bourges H., Casanueva E., Rosado J.L. (2009). Recomendaciones de Ingestión de Nutrimentos Para La Población Mexicana.

[34] Barquera S., Hernández-Barrera L., Campos-Nonato I., Espinosa J., Flores M., J A.B., Rivera J.A. (2009). Energy and nutrient consumption in adults: Analysis of the mexican national health and nutrition survey 2006. Salud Publica Mex..

[35] Denova-Gutiérrez E., Clark P., Muñoz-Aguirre P., Flores M., Talavera J.O., Chico-Barba L.G., Rivas R., Ramírez P., Salmerón J. (2016). Dietary patterns are associated with calcium and vitamin D intake in an adult Mexican population. Nutr. Hosp..

[36] Ma Z.J., Yamaguchi M. (2001). Role of endogenous zinc in the enhancement of bone protein synthesis associated with bone growth of newborn rats. J. Bone Miner. Metab..

[37] Lowe N.M., Fraser W.D., Jackson M. (2002). Is there a potential therapeutic value of copper and zinc for osteoporosis?. Proc. Nutr. Soc..

[38] Razmandeh R., Nasli-Esfahani E., Heydarpour R., Faridbod F., Ganjali M.R., Norouzi P., Larijani B., Khoda-Amorzideh D. (2014). Association of Zinc, Copper and Magnesium with bone mineral density in Iranian postmenopausal women—A case control study. J. Diabetes Metab. Disord..

[39] Bolan C.D., Cecco S.A., Wesley R.A., Horne M., Yau Y.Y., Remaley A.T., Childs R.W., Barrett A.J., Rehak N.N., Leitman S.F. (2002). Controlled study of citrate effects and response to IV calcium administration during allogeneic peripheral blood progenitor cell donation. Transfusion.

[40] Vasu S., Leitman S.F., Tisdale J.F., Hsieh M.M., Childs R.W., Barrett A.J., Fowler D.H., Bishop M.R., Kang E.M., Malech H.L. (2008). Donor demographic and laboratory predictors of allogeneic peripheral blood stem cell mobilization in an ethnically diverse population. Blood.

[41] Mercan D., Bastin G., Lambermont M., Dupont E. (1997). Importance of ionized magnesium measurement for monitoring of citrate- anticoagulated plateletpheresis. Transfusion.

[42] Toffaletti J., Nissenson R., Endres D., McGarry E., Mogollon G. (1985). Influence of continuous infusion of citrate on responses of immunoreactive parathyroid hormone, calcium and magnesium components, and other electrolytes in normal adults during plateletapheresis. J. Clin. Endocrinol. Metab..

[43] Peiris A., Youssef D., Grant W. (2012). Secondary hyperparathyroidism: Benign bystander or culpable contributor to adverse health outcomes?. South Med. J..

[44] Vetter T., Lohse M.J. (2002). Magnesium and the parathyroid. Curr. Opin. Nephrol. Hypertens..

